# Comparison of 116 Radiosurgery Treatment Plans for Multi-Leaf and Cone Collimator on a Varian Edge Linac: Are Cones Superior in the Daily Routine?

**DOI:** 10.3390/life13041020

**Published:** 2023-04-15

**Authors:** Adlan Čehobašić, Josip Paladino, Hrvoje Kaučić, Ana Mišir-Krpan, Vanda Leipold, Mihaela Mlinarić, Domagoj Kosmina, Andreas Mack, Dragan Schwarz, Sunčana Divošević, Ivana Alerić

**Affiliations:** 1 Specijalna Bolnica Radiochirurgia Zagreb, Ulica Dr. Franje Tuđmana 4, 10431 Sveta Nedelja, Croatia; 2 Medicinski Fakultet Osijek, Sveučilište Josipa Jurja Strossmayera u Osijeku, Josipa Huttlera 4, 31000 Osijek, Croatia; 3 Medicinski Fakultet, Sveučilište u Zagrebu, Šalata 3, 10000 Zagreb, Croatia; 4 Swiss NeuroRadiosurgery Center, Bürglistrasse 29, 8002 Zürich, Switzerland; 5 Medicinski Fakultet, Sveučilišta u Rijeci, Braće Branchetta 20/1, 51000 Rijeka, Croatia; 6 Fakultet za Dentalnu Medicinu i Zdravstvo Osijek, Sveučilište Josipa Jurja Strossmayera u Osijeku, Crkvena Ulica 21, 31000 Osijek, Croatia

**Keywords:** radiation therapy, brain metastasis, multi-leaf collimator, conical collimator, treatment plan, robustness test, QA measurements

## Abstract

Delivering focused radiation doses via linear accelerators is a crucial component of stereotactic radiosurgery (SRS) for brain metastases. The Varian Edge linear accelerator provides highly conformal radiation therapy through a high-definition multi-leaf collimator (HD120 MLC) and conical collimator (CC). HD120 MLC adapts to the shape of the target volume using movable tungsten leaves, while CC has a block of conical shape (cones). CC in SRS treatments of small brain metastases is preferred due to its mechanical stability and steeper dose fall-off, potentially sparing organs at risk (OARs) and the brain better than HD120 MLC. This study aims to determine if CC offers significant advantages over HD120 MLC for SRS treatments. For 116 metastatic lesions, CC and HD120 MLC treatment plans were created in Varian Eclipse TPS and compared based on various dose parameters, robustness tests, and QA measurements. The results indicate that CC provides no significant advantages over HD120 MLC, except for slight, clinically insignificant benefits in brain sparing and dose fall-off for the smallest lesions. HD120 MLC outperforms CC in almost every aspect, making it a better choice for irradiating brain metastases with 0.1 cm^3^ or higher volumes.

## 1. Introduction

Brain metastases, traditionally seen as a terminal phase of cancer, generally affect 10-40% of patients with malignancies [1,2,3,4]. Despite their poor prognosis, treatment modalities and technological advances have improved clinical outcomes for patients with brain metastases [3,4]. Stereotactic radiosurgery (SRS) is a form of radiation therapy that involves delivering high doses of focused radiation to small, well-defined structures (lesions, target volumes) using tight beams of ionizing radiation such as X-rays, gamma rays, or protons. SRS aims to halt the proliferation of benign tumors or permanently destroy tumor tissue without an invasive approach while minimizing damage to surrounding healthy tissue. SRS can be administered in a single session or over a series of up to five fractions [5,6]. The efficacy of stereotactic radiosurgery (SRS) for brain metastases has been demonstrated to provide good local control (LC) at the one-year mark; however, its effectiveness is heavily influenced by the prescribed dosage and fractionation regimen. Studies have indicated that brain metastases receiving over 21 Gy in a single fraction can achieve LC rates between 85–92%, whereas lower doses below 15 Gy result in poor LC rates, less than 50% [7,8,9,10]. Notably, the achieved LC rates of SRS treatments are similar to those of surgical interventions [11,12]. Warsi et al. have demonstrated the cost-effectiveness of SRS when compared to surgical interventions, as SRS has shorter treatment durations and fewer required hospitalizations [13]. Similarly, Lester-Coll et al. have shown that SRS is more cost-effective than whole-brain radiation therapy [14].

Radionecrosis can present itself with diverse symptoms, such as brain inflammation and swelling, seizures, alterations in cognition, and changes in motor function or coordination [15].

Critical doses for developing radionecrosis are generally considered 10–12 Gy in a single fraction [16,17,18]. It is crucial to achieve high dose conformality, reducing the volume of healthy tissue receiving high doses of radiation to minimize the risk of side effects.

A linear accelerator’s photon beams can be focused using one or two sets of jaws and shaped using either a multileaf collimator (MLC) or conical collimator (CC). The MLC shapes the irradiation field using movable tungsten leaves, allowing it to conform to the target volume (PTV) shape and reduce the dose to healthy tissue. Conversely, CC consists of a block with fixed conical shapes (cones) that are mechanically stable, generate less scatter than MLCs, and have a steeper dose gradient [19].

In radiation therapy, the degree of sharpness of the dose gradient is determined primarily by the physical penumbra. This region of the dose profile, located at the edge of the radiation field, exhibits a rapid decrease in dose with increasing distance from the beam axis. The steepness of the penumbra is affected by three key factors: transmission, geometry and scatter. The contribution of transmission to the penumbra is attributed to the portion of radiation passing through the secondary collimator, which may be a cone or MLC. The geometric contribution arises from the shape of the secondary collimation and its impact on source occlusion or the degree to which primary radiation from the source is unobstructed by secondary collimation. The scatter contribution results from the scattering of doses, primarily within the patient. The steeper dose fall-off observed for CC is due to the negligible cone transmission, lower levels of direct primary photons, and reduced scattering effects within the patient [19,20].

However, both MLC and CC have limitations. The mechanical stability of MLC is not the sole limitation of their use in radiation therapy. Additionally, modeling MLC and calculating doses can be challenging in treatment planning systems (TPSs). For example, the minimum field size that can be calculated in Eclipse (Varian Medical Systems, Palo Alto, California) is 1 × 1 cm, which may not meet clinical standards for dose calculation. The non-small field sizes utilized in radiation therapy treatments span from 3 × 3 cm^2^ to 40 × 40 cm^2^ [21]. For intricate treatment techniques such as volumetric modulated arc therapy (VMAT), TG-106 recommends acquiring commissioning beam data across a broader range of 1 × 1 cm^2^ to 40 × 40 cm^2^ [22]. The smallest field size that can be imported into Varian Eclipse Beam Configuration is 2 × 2 cm^2^ for percent depth dose (PDD) and profile curves. However, the output factor table can be populated for 1 × 1 cm^2^ field sizes [23]. In the context of the HD120 MLC, a model-based algorithm is used for dose calculation. In clinical scenarios, the lesions may have minimal volumes, as small as 0.1 cm^3^ with a radius of 2.8 mm, and MLC-formed fields may be only a few millimeters in size. When modeling fields smaller than 1 × 1 cm^2^, the dosimetric leaf gap is the only parameter utilized for model adjustment, which leads to increased uncertainty in the calculated dose [24]. Consequently, the TPS may significantly overestimate or underestimate the calculated dose, depending on the acquired commissioning data, which is mainly influenced by the detectors used for acquiring commissioning data [25,26].

The Eclipse Cone Dose Calculation (ECDC) algorithm utilizes three measurements: tissue-maximum ratio, off-axis ratio, and cone output factor. However, the ECDC algorithm has several limitations. It does not account for tissue heterogeneity, assumes that the beam axis is normal to the patient surface (without correction for oblique beam incidence), and does not consider backscatter. Additionally, the off-axis ratio is independent of depth, and arc beams are approximated as evenly distributed static fields. To calculate the average tissue-maximum ratio for the arc, the ECDC draws static beams at user-specified arc increments and then averages the tissue-maximum ratio from all of these beams to determine an average value for dosimetry calculations [23].

While the MLC is a standard component in linear accelerator systems, the CC is an expensive optional accessory purchased separately. The comparison of MLC and CC is a well-established topic in the literature, with numerous studies providing different perspectives on using CC in radiation therapy.

To name a few, Borzov et al. (2019) performed a dosimetric characterization of Elekta’s CC, and the results show that it exhibited lower leakage and a sharper penumbra compared to MLC [24]. Li et al. (2016) conducted a treatment-planning study to determine the optimal Linac-based SRS strategy by comparing the aperture effects of cone and MLC-based plans for small target treatment. The study found that single arc plans reduced V12Gy volumes for CC [25]. Additionally, Xu et al. (2019) retrospectively compared the neurotoxicities of 63 patients (14 cones, 49 MLC) with small arteriovenous malformations over 970 days. The study found that MLC plans had higher 4 Gy and 12 Gy volumes, associated with increased neurotoxicities [26].

Despite the abundant literature on the topic, a comprehensive understanding of the actual benefits of CC in clinical practice still needs to be achieved. To bridge this knowledge gap, we conducted a retrospective study of 116 cases of brain metastases. Our analysis involved calculating treatment plans for every brain metastasis for CC and HD120 MLC with the same or similar beam geometry and comparing dose distributions inside and outside the target volumes, treatment times, and the number of monitor units. Absorbed doses in organs at risk (OARs) were also compared. For the brain, 10 Gy and 5 Gy volumes were compared, while for all other OARs, only maximum doses were considered. Both methods were tested for small movements, and dosimetric measurements were performed using a StereoPhan phantom and 2D dosimetric array SRS MapCheck [27,28,29,30]. We also addressed other potential clinical issues. The findings of this study are expected to offer valuable insights into the clinical merits and drawbacks of CC, thereby contributing to a more informed understanding of its utility in radiotherapy practice.

## 2. Materials and Methods

### 2.1. Selection

From the Aria© system (Varian Medical Systems, Palo Alto, CA, USA), 116 brain metastases were selected for patients who received SRS at Specijalna Bolnica Radiochirurgia Zagreb between 2017 and 2021. Brain metastases were contoured on 1 mm CT slice thickness as clinical target volume (CTV), and a 1 mm isotropic margin was added for creating PTV. The selection criteria included PTV volumes between 0.1 and 6.5 cm^3^, a PTV shape as close to a sphere as possible, and no overlap with organs at risk (OARs) such as the optical pathway, chiasm and brainstem. The PTVs were divided into four groups based on volume: group 1: 0.1–0.5 cm^3^, group 2: 0.6–0.9 cm^3^, group 3: 1.0–2.5 cm^3^, and group 4: 2.6–6.5 cm^3^. The numbers of PTVs in groups were: 35 for group 1, 35 for group 2, 35 for group 3 and 11 for group 4.

### 2.2. Treatment Plans

The study employed the following planning parameters: a 6X FFF energy, RapidArc (VMAT) technique, a single fraction, and normalization of 25 Gy covering 99.5% of the planning target volume (PTV) for groups 1–2, and 25 Gy covering 95% of the PTV for groups 3–4.

Treatment plans utilizing a HD120 MLC were based on a single isocenter with a fixed field size 2 × 2 cm^2^, while plans using CC employed one to ten isocenters. Inspired by Varian’s hyperarc technique, the single isocenter arcs for any collimator comprised one full arc with a couch angle of 0 and nine partial arcs with identical start and stop angles and couch rotations. The multi-isocentric arcs, specific to the CC plans, were composed of four non-coplanar partial arcs individually selected for each isocenter.

The HD120 MLC plans were optimized using the Photon Optimizer (PO 16.1.0) at a resolution of 1.25 mm. The PO algorithm accounted for corrections of inhomogeneities and air cavities. Due to the limitations of the PO algorithm, higher resolutions are not possible. The final dose calculations were performed using the AcurosXB (16.1, dose to medium) algorithm with a resolution of 1.00 mm, as the TPS Varian Eclipse does not support finer resolutions.

The CC’s planning module lacks an optimization algorithm. The optimization and final dose calculation procedures rely on the ECDC (16.1) algorithm. The only difference is that the dose is calculated only in three currently intersected planes during optimization. A new dose calculation is initiated when a different cross-section is chosen. Therefore, the dose optimization was performed through isocenter placement and movement adjustments, cone size selection, modifications of arc geometry, and couch rotation. An arc resolution of 1 degree, a dose matrix resolution of 1 mm and a slice interval of 1 mm were used for optimization. Final dose calculations were performed using an arc angle resolution of 1 degree, dose matrix resolution of 0.6 mm, and dose slice interval of 0.6 mm. A slight discrepancy in the dose grid resolution between the HD120 MLC and CC plans can be observed. The impact of this discrepancy on the final results is anticipated to be minimal, and it will be addressed in the discussion section of the manuscript. It is important to note that none of these plans were used for patient treatment.

### 2.3. Comparison Parameters

Uncertainties in the minimum, maximum and mean doses within PTVs have been noted. To address this, the International Commission on Radiation Units and Measurements (ICRU) has recommended in their Report 83 (ICRU 83) the use of dose coverage encompassing 2% and 98% of the PTV instead of relying on maximum and minimum doses, respectively. Furthermore, the report suggests using the median dose instead of the mean [31].

The comparison of plans involved the evaluation of the following parameters for the planning target volume (PTV): conformity index (CI), homogeneity index (HI), dose gradient index (DGI) and Paddick index (PI), as well as minimum, maximum and mean dose. For doses outside of the PTV, the parameters considered were dose volumes of 25 Gy, 12.5 Gy, 10 Gy, and 5 Gy around the PTV, 5 Gy and 10 Gy brain dose volumes, total number of monitor units (MU) and maximum dose for organs at risk (OARs).

The doses and volumes were obtained from the Varian Eclipse treatment planning system. However, it was necessary to compute the dose parameters using the following method [32,33,34,35,36,37,38]:

(1) $$CI=\frac{Prescribed\;Dose\;Volume}{PTV\;Volume}$$

(2) $$HI=\frac{\left(D_{2}-D_{98}\right)}{D_{50}}$$

(3) $$DGI=\frac{50\%\;isodose\;volume}{Prescribed\;dose\;volume}$$

(4) $$PI=\frac{PTV\;volume\times PTV\;volume}{PTV\;volume\times Prescribed\;dose\;volume}$$

### 2.4. Robustness Test

To evaluate the sensitivity of both HD120 MLC and CC plans to small isocenter movements, isocenters were shifted by ±1 mm in all three axes. In clinical practice, the patient’s head is immobilized using a couch headrest and thermoplastic mask; however, perfect immobilization cannot be guaranteed, and small movements of less than 1 mm may occur. Therefore, 1 mm isocenter shifts were considered as the worst-case scenario. While test plans for HD120 MLC could be automatically created and calculated using the Varian Eclipse TPS, CC test plans had to be created manually. Robustness test plans were created only for group one and single isocenter plans, and the rationale behind this decision will be discussed in the manuscript’s discussion section. The subsequent analysis only considers the direction of the maximum shift and its impact on the PTV dose coverage.

### 2.5. QA Measurements

The Varian Eclipse TPS includes functionality for generating quality assurance (QA) plans from pre-existing treatment plans, albeit restricted to HD120 MLC plans. For CC plans, the manual creation of QA plans is required. Given the complex nature of CC plans involving multiple table rotations and isocenters, generating a verification QA plan is arduous and susceptible to errors. Because of these challenges, simpler QA plans were used to evaluate both algorithms.

Sun Nuclear’s SRS MapCheck 2D dosimetric array was inserted into the StereoPHAN phantom for end-to-end evaluations (SunNuclear Corporation, Melbourne, FL, USA), with CT scans obtained at a 1 mm slice thickness. The SRS MapCheck system comprises 1013 SunPoint 2 diode detectors, featuring an active detector area of 0.48 × 0.48 mm and a detector spacing of 2.47 mm over an array size of 77 × 77 mm.

For QA plans without table rotations, spherical PTVs with diameters of 4 mm, 5 mm, 7.5 mm, 10 mm, 12 mm, 15 mm and 17.5 mm were delineated at the position of the central detector. Treatment plans for both collimators had identical geometries, consisting of a single 180-degree partial arc commencing at an angle of 270 degrees and ending at 90 degrees. All plans were normalized to 1 Gy, encompassing 99.5% of the PTV. For HD120 MLC plans, the jaws were fixed at a 2 × 2 cm field while optimizing dose delivery through the leaves, while the CC plans comprised a single cone for each PTV size.

QA measurements using table rotations were conducted for three distinct PTVs of varying volumes. The PTVs had diameters of 3.9 mm, 7 mm and 11.1 mm, with corresponding volumes of 0.03 cm^3^, 0.18 cm^3^ and 0.73 cm^3^, respectively. The PTVs were contoured 3 cm above the central detector of the SRS MapCheck along the central axis to ensure adequate space and minimize collision risks between the cone and phantom.

Three partial arcs were generated for the QA plan, each with specific start and stop angles and couch rotations. Arc 1 spanned from a start angle of 270 degrees to a stop angle of 90 degrees, with a couch rotation of 0 degrees. Arc 2 ranged from a start angle of 60 degrees to a stop angle of 180.1 degrees, with a couch rotation of 25 degrees. Finally, Arc 3 covered from a start angle of 180.1 degrees to a stop angle of 30 degrees, with a couch rotation of 50 degrees. A CBCT scan was performed before each measurement to verify the accurate positioning of the phantom. Since all group treatment plans shared the same isocenter, further CBCT checks during measurements were unnecessary.

After the measurements, the gamma analysis criteria were 1 mm 1%, with a dose threshold of 1%.

### 2.6. Statistics

The normality of all values was assessed using the Kolmogorov–Smirnov test, with results showing *p* < 0.005, indicating non-normal distribution. The Wilcoxon signed-rank test (matched pairs) was used for all subsequent comparative tests. The appropriate sample size was determined using G*Power software (v. 3.1), with a power of 0.95, alpha of 0.05, and effect size of 0.59, yielding a required sample size of 35 pairs of treatment plans in each group [39,40]. All null hypotheses were formulated assuming that distributions across modalities (HD120 MLC and CC) were equivalent. A significance level of *p* < 0.05 indicated that the null hypothesis was rejected and that significant differences existed between the two sets of plans. Conversely, a significance level of *p* > 0.05 indicated that the null hypothesis was upheld and that no significant differences were present.

## 3. Results

### 3.1. Dose Distributions in PTV

**Figure 1:**
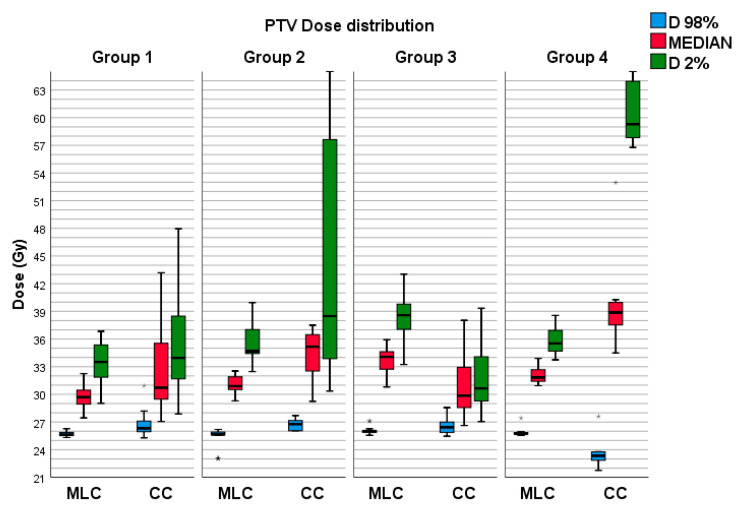
Distribution of PTV D_98%_, D_2%_ and median doses for each collimator and group. The outliers have been denoted by the asterisk symbol (*).

The median, D_98%_ and D_2%_ doses were extracted from treatment plans in Varian Eclipse TPS to obtain PTV dos distributions values. The resulting data consisted of mean values and corresponding standard deviations of D_98%_, D_2%_ and median doses for each PTV within all four groups, as detailed in Table 1. A graphical representation of the distribution of these results can be found in Figure 1. Statistical analysis results shows statistically significant differences for all parameters except D_2%_ in group 1 (*p* = 0.128)

### 3.2. Dose Fall-Off

**Figure 2:**
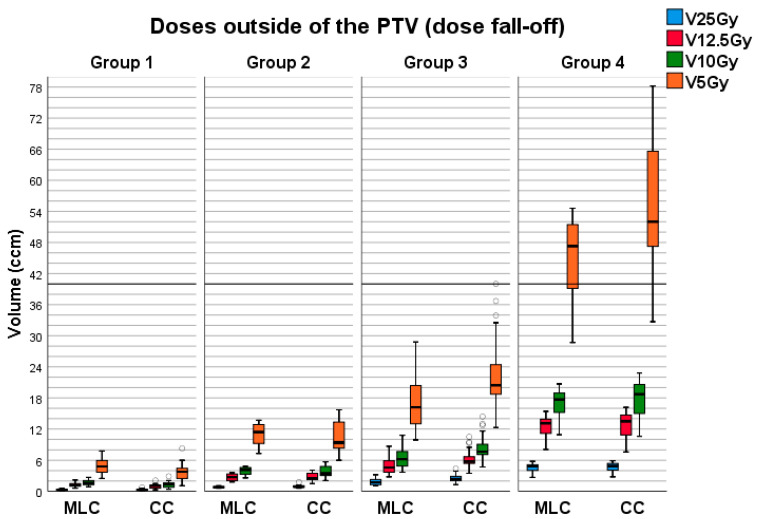
Distributions of fall-off doses for every collimator and group. The outliers have been symbolized by the circular icon (°).

The dose fall-off, characterized by dose distributions outside of the PTV, was also exported from the TPS. The desired isodose lines, including 25 Gy, 12.5 Gy, 10 Gy and 5 Gy, were transformed into DICOM RT structures using Varian Eclipse TPS. The resulting volumes of these structures for each plan and collimator within every group and collimator are presented as mean values with corresponding standard deviations in Table 2. A visual representation of the distribution of these results can be found in Figure 2. Statistical analysis did not reveal statistically significant differences only in groups 2 and 4 for V_12.5Gy,_ V_10Gy_ and V_5Gy_.

### 3.3. Dose Parameters

**Figure 3:**
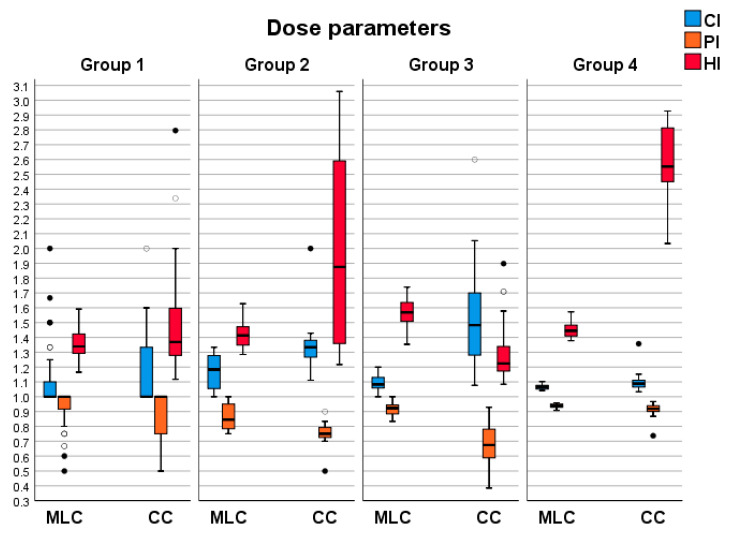
Distributions of PTV parameters for both collimators across all groups. The transparent and black circular icons symbolize the outliers. Different color fills indicate there are outliers in more than one plan.

Dose parameters were determined based on the data collected for each treatment plan. The Varian Eclipse TPS was used to extract mean, minimum and maximum doses to obtain PTV dose distribution values. At the same time, formulas in Section 2.3, Equations (1)–(4), were utilized to calculate other dose parameters. The resulting dataset included mean values and corresponding standard deviations of minimum, maximum and mean doses for each PTV in all four groups, as presented in Table 3. A graphical representation of the distribution of these results is depicted in Figure 3. Statistical analysis did not reveal significant differences in the parameters CI (group 4, *p* = 0.091), HI (groups 1 and 2, *p* = 0.422 and *p* = 0.341), PI (group 4, *p* = 0.091).

### 3.4. Absorbed Doses in the Brain

**Figure 4:**
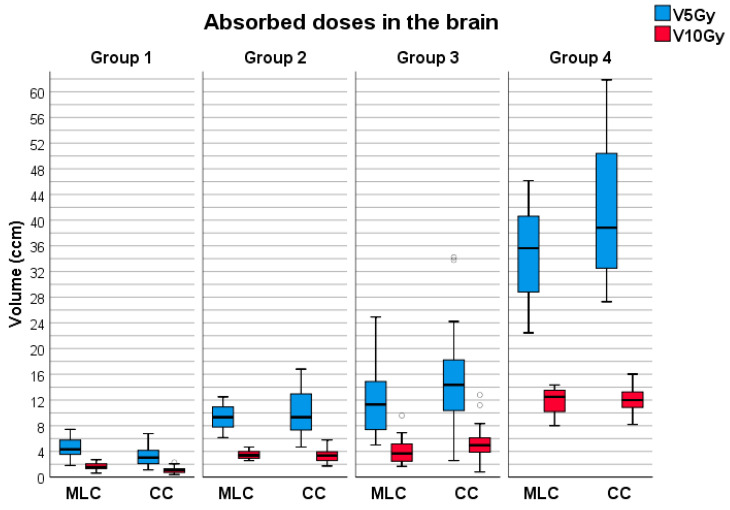
Brain dose distributions for both collimators across all groups. The outliers have been symbolized by the circular icon (°).

Absorbed doses in the brain were obtained from Varian Eclipse TPS. The lowest dose associated with the development of radionecrosis in the brain is 10 Gy, while 5 Gy is half the dose, indicating potential for future irradiations near selected PTVs. The resulting dataset included mean values and corresponding standard deviations of volumes of 10 Gy and 5 Gy doses absorbed in the brain across all groups. Table 4 presents the mean values with standard deviations. A graphical representation of the distribution of these results is depicted in Figure 4. Statistical analysis did not reveal significant differences in the following parameters for Group 2: V_5Gy_ (*p* = 0.237), V_10Gy_ (*p* = 0.140), and for Group 4: V_5Gy_ (*p* = 0.286), V_10Gy_ (*p* = 0.345).

### 3.5. Maximum Doses of OARs

**Figure 5:**
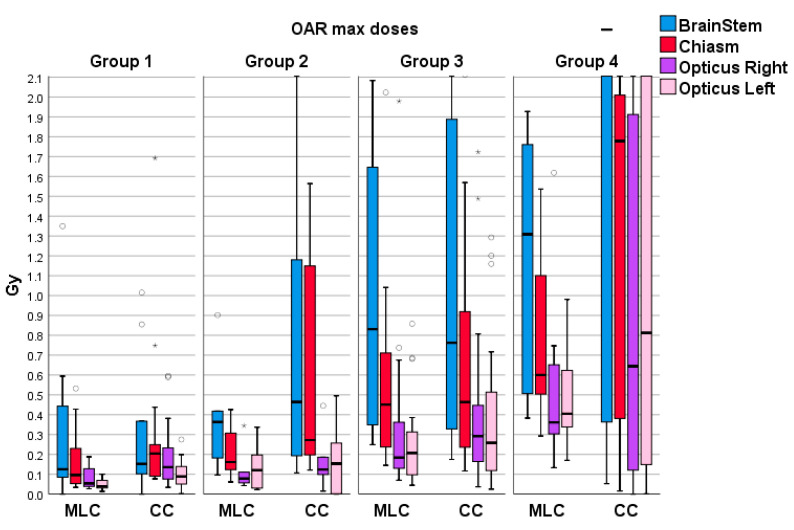
Maximum dose distributions for four OARs with highest maximum doses for both collimators across all groups. The outliers have been symbolized by the circular (°) and asterisk symbols (*). Different symbols indicate there are outliers in more than one plan.

Table 5 represents the median values (in Gy) of maximum doses for each OAR in the dataset. The rationale is that the minimum OAR doses are mostly 0 Gy or only a few centigrays. At the same time, almost every OAR contains one significant outlier that dramatically affects the mean dose and standard deviation, resulting in a higher standard deviation than the mean value. Therefore, the decision was made to show more representative values by presenting the median of the maximum doses. Figure 5 displays the distributions of maximum doses for the four OARs with the highest absorbed dose maximums.

### 3.6. Robustness Test

**Figure 6:**
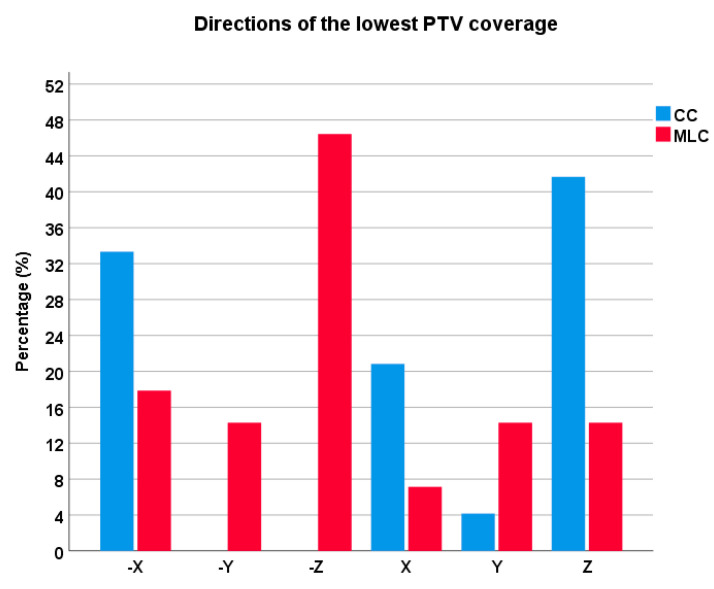
Percentage of PTVs distributed along axes and directions. Isocenter shift ±1 mm on axes are displayed as ±X, ±Y and ± Z.

Table 6 displays the results of robustness tests for Group 1 only. The isocenters in the plans were shifted ±1 mm in all three axes. As isocenter shifts affect PTV coverage, the lowest PTV coverage from each plan for each collimator was included in the dataset. The dataset in the first part of Table 6 represents the lowest PTV coverages for both collimators. The minimum coverage means the lowest obtained coverage in the group, while the maximum is the maximum lowest PTV coverage. Figure 6 illustrates the frequency distributions of directions exhibiting the lowest PTV coverage.

In the second part of Table 6, a comparison is made by looking at treatment plan pairs where each PTV had two sets of isocenter shifts for both collimators. Comparisons consider only differences between plans in one pair. The number of plans with the higher lowest PTV coverage indicates how many times across Group 1 one collimator had higher PTV coverage than the other for the same PTV. The highest PTV coverage difference between pairs shows the highest distinction in PTV coverage for one pair. When HD120 MLC had higher PTV coverage, the highest difference between HD120 MLC and CC for the same PTV was 25.97%, while CC had higher PTV coverage with a difference of 4.41%.

The number of plans with the lowest PTV coverage valued at least 95% indicates how many times PTV coverage was slightly affected by the isocenter shift. In contrast, the range of 90–94.99% means how many times plans were moderately affected by shifts. The number of plans with the lowest PTV coverage below 90% indicates how often treatment plans were significantly affected by isocenter shifts.

### 3.7. QA Measurements

The results of the QA measurements are presented in terms of the passing rates of gamma analysis for criteria 1 mm 1% with a dose threshold of 1%. The measurements were repeated five times, and no deviations from the results were observed, as all measurements yielded identical outcomes. Table 7 shows the gamma analysis scores of the measurements for seven distinct PTV diameters for both collimators without couch rotations. In contrast, Table 8 illustrates the gamma analysis passing rates for three PTV volumes for both collimators with two couch rotations.

## 4. Discussion

Before comparing treatment plans, it is crucial to address two of the most significant challenges associated with CC: imaging and planning software.

For any radiosurgery treatments, the patient must be as close as possible to the same position as planned. The position is verified through imaging, with CBCT mandatory for radiosurgery treatment. However, when the CC is attached to the linear accelerator’s head, CBCT is disabled, along with all automatic couch motions. To perform CBCT, the CC must be removed from the linear accelerator, and after the procedure, it must be reattached. However, in certain situations, the couch (with the patient) must be manually moved to make room for the CC mount and then manually shifted back to its original position. For CC treatment plans using a single isocenter and two couch rotations, radiation therapy technologists (RTTs) must enter linac’s vault at least once for CBCT and twice for the couch rotations. This results in significantly prolonged treatment times and increases the risk of errors. For multiple isocenters, the number of mandatory vault entries, treatment prolongation and potential for mistakes escalate rapidly.

Treatment planning quality relies heavily on software capabilities, and Varian’s cone planning software appears incomplete. CC treatment planning utilizes a forward planning approach without inverse planning options to shape the fluence map or establish maximum dose objectives. Consequently, CC is almost unusable for prescriptions requiring homogeneous dose distribution.

All four groups exhibit similar dose behavior inside the PTV. While D_98%_, median, and D_2%_ are higher in CC plans, HD120 MLC plans possess higher minimum dose values and cold spots at least 2 Gy greater than CC plans. CC plans exhibit higher D_2%_ and median doses in most scenarios for two reasons. Firstly, multi-isocentric plans experience high-dose hotspots due to dose superposition from every isocenter. Secondly, in single-isocentric CC plans, if the cone diameter cannot cover the entire PTV and the next available diameter extends too far outside the PTV, the prescription dose must be increased until normalization of 25 Gy is attained to achieve dose coverage.

In groups 1, 2, and 4, CC plans had a 9%, 14%, and 31% higher median dose, respectively, and the maximum dose difference was also higher, with values of 7%, 21%, and 68%. Group 3, however, showed an exception with a 6% higher median dose and 18% higher maximum dose in HD120 MLC plans. PTV geometry influenced this change in group 3, as PTV tended to grow in one direction more than the other, which is the worst-case scenario for CC. Cones that can be used are determined by the smallest plane, and CC using multiple isocenters with small cones may not achieve high maximum doses as in other groups. Unfortunately, Varian Cone Planning lacks an inverse planning option to restrict high hotspots inside PTV, which is common with CC.

Similarly to the previous results, dose parameters show specific behavior across all groups. While HD120 MLC can generally create plans with lower CI than CC, usually with a mean value around 1.10, both methods can struggle and create plans with high CI. High CI in HD120 MLC plans is associated only with the inability to conform the prescription dose to the PTV shape due to imperfections. For CC plans, it is also associated with lower PTV coverage. Despite their disadvantage in forward planning, CC plans can also create plans with plans with low CI.

For group 1, CC plans have only a 6% higher CI, while for groups 2 and 3, the difference is 18% and 40%, respectively, and only 4% for group 4. PI values are accordant to CI. In all groups, PI values are over 0.9 for HD120 MLC plans, while CC plans are 0.81–0.90. The most significant differences are in groups 2 and 3, 12% and 13%, respectively, for the reasons explained earlier.

HI, defined as the ratio of the difference between D_2%_ and D_98%_ doses in PTV and the D_50%_ dose, is significantly higher in CC plans, as those plans have higher maximum PTV doses.

DGI, defined as the ratio of volumes of 50% and 100% isodoses, is lower in CC plans. The differences across groups 1–4 are 55%, 4%, 10%, and 1% lower DGI in CC plans.

In groups 2–4, the HD120 MLC achieved higher dose control outside the PTV, resulting in a better dose fall-off than the CC. Lower dose control in CC plans was not the consequence of the multi-isocentric approach, as more isocenters only help to achieve higher dose conformity without affecting 12.5 Gy and 10 Gy isodose volumes. For 5 Gy, it remains to be seen. However, in group 1, the situation changed. Statistical analysis revealed significant differences across groups for 25 Gy, 10 Gy, and 5 Gy volumes, while 12.5 Gy did not show significant differences. CC plans delivered a slightly higher mean 25 Gy volume (0.04 cm^3^) with a maximum of 0.2 cm^3^ higher, but the steeper dose fall-off in CC plans finally showed its effect. All values for volumes below 25 Gy were lower in CC plans, with a difference of 0.3 cm^3^ for V12.5 Gy, 0.43 cm^3^ for V10Gy, and 1.24 cm^3^ for V5Gy. Although these differences can impact statistical significance for small values, they are negligible in clinical practice.

DGI values indicate a steeper dose fall-off, which can improve the sparing of brain tissue and other organs at risk. However, the advantage of CC in sparing brain tissue was slight for group 1, while HD120 MLC provided better results for the other groups.

Statistical analysis showed no significant differences in datasets across groups 2 and 4. In HD120 MLC plans, the V_5Gy_ was lower by 0.92 cm^3^ for group 2, 3.28 cm^3^ for group 3, and 6.59 cm^3^ for group 4. Similarly, the V_10Gy_ was lower by 0.04 cm^3^, 1.27 cm^3^, and 0.47 cm^3^ for groups 2, 3, and 4, respectively.

In group 1 and 3, statistical analysis revealed significant differences across the groups. The volume of brain tissue in group 1 receiving 5 Gy ranged from 1.81 to 7.44 cm^3^ with a mean value of 4.54 Gy for HD120 MLC, while for CC plans, the absorbed dose for the same radiation doses ranged from 1.13 to 6.78 cm^3^ with a mean value of 3.2 cm^3^. The volume of brain tissue receiving 10 Gy ranged from 0.62 to 2.72 cm^3^ with a mean value of 1.63 cm^3^ for HD120 MLC and 0.38 to 2.29 cm^3^ with a mean value of 1.09 cm^3^ for CC plans. The results indicate that the difference could be even higher for smaller volumes. However, these findings should be interpreted in light of previous research showing that radiation doses of 10 Gy and 12 Gy are critical for potential radionecrosis, with no more than 10 cm^3^ of brain tissue receiving these doses. Although the numerical differences between the collimators are significant, a 0.5 cm^3^ higher dose absorbed in the brain in HD120 MLC plans should be an acceptable trade-off when considering the CC’s imaging issues mentioned earlier.

In cases of multiple metastases located near each other, HD120 MLC can provide a single isocenter that encompasses all PTVs. This setup allows for the delivery of prescribed doses to every PTV while minimizing the dose between PTVs using various dose constraints and generalized equivalent uniform dose (gEUD) constraints. Additionally, helper contours can be created to shape the dose around the PTVs. In contrast, CC planning requires a separate isocenter for each PTV. In such a setup, the inability to shape the dose fluence in Cone Planning software would cause all low doses in CC plans to sum, resulting in much higher volumes of 5 Gy and 10 Gy compared to HD120 MLC.

When metastases are far apart, and a few separate isocenters are needed, CC plans may result in lower doses absorbed in the brain. However, considering technical issues, HD120 MLC plans can be delivered within an hour on the same day, while CC plans take at least twice the time.

It should be noted that the prescribed dose of 25 Gy used in this study is not commonly used for brain metastases. Prescribed doses typically range from 16 Gy to 24 Gy, and lower doses result in fewer differences between plans. Thus, 25 Gy is likely the least favorable prescription for the clinical treatment of brain metastases.

Absorbed dose OARs differ from the trend of absorbed dose in the brain. OARs are located further away from the PTV and receive lower radiation doses. While this is favorable for sparing these organs, it poses a challenge for CC planning. The optimizer in the HD120 MLC plans can include numerous dose constraints to shape the dose fluence in the plan. However, optimizing the low radiation doses is difficult when the OARs are positioned far from the PTV. Very high-priority dose constraints must be placed in the optimizing software, which can violate the achieved radiation dose distributions inside and outside the PTV.

The results for doses absorbed by OARs varied between plans. While the statistical analysis showed statistically significant differences for every OAR across at least one group, it is hard to notice significant differences across organs except for a few outliers that influenced the statistics. Results are not following any trend, so interpreting the results solely on the numbers from Table 5 is difficult. Based on the results obtained, it can be inferred that the steeper dose fall-off observed in CC is not a significant factor in sparing the OARs.

Treatment time and monitor units are variables that cannot be observed separately. Higher monitor units in a treatment plan typically result in longer treatment times. In this study, for the HD120 MLC plans, the monitor units and treatment time remained relatively constant across all four groups, with a range of 5500 to 7600 monitor units and a treatment time of 256 to 684 s. A continuous number of MUs indicates that the clinical treatment could be completed within 15 min. However, the number of monitor units needed for CC plans is unpredictable and depends on the number of isocenters and cone size. Smaller cone diameters require more monitor units and, consequently, more time to deliver the same radiation dose.

In some cases, CC plans needed as low as 3500 monitor units, while up to 9700 monitor units were needed in others. Multi-isocentric CC plans can require up to 20,000 or even 30,000 monitor units. While TPS may estimate that CC plans require less time than HD120 MLC plans, CBCT time and manual patient shifts are not taken into account. A simple geometry plan with a single full arc may have a shorter beam-on time for CC plans than HD120 MLC plans, but the dose distribution may not be as good as HD120 MLC plans.

The test involved shifting the isocenter by ±1 mm along each axis, but only for single-isocentric plans and group 1. This decision was made based on the results of this study, which show that CC should only be used for small spherical targets on a single isocenter. While CC can be used for other cases, the study results indicate that HD120 MLC produces better results with more straightforward treatment.

The robustness test revealed interesting results, showing that HD120 MLC is less susceptible to problems with PTV coverage than CC. Comparing PTV coverages from treatment plan pairs, CC had higher PTV coverage in only seven cases, with a maximum difference of 4.41%. In contrast, HD120 MLC had higher PTV coverage in the remaining 28 cases, with the highest difference being 25.97%. Moreover, isocenter shifts in HD120 MLC plans will either significantly impact PTV coverage or have almost no impact, whereas CC plans were substantially more sensitive to isocenter shifts. Specifically, HD120 MLC had 23 cases with PTV coverage over 95% and 12 cases below 90%, while CC had only one case with PTV coverage over 95%, nine cases over 90%, and the rest were cases below 90%.

In summary, while isocenter shifts can lower PTV coverage in HD120 MLC plans, CC plans are much more vulnerable to such shifts. If HD120 MLC achieves excellent PTV dose coverage, CC’s coverage will be significantly worse. Conversely, if HD120 MLC’s dose coverage is significantly reduced, CC’s coverage will be equally inadequate or slightly better.

Additionally, the study found no conclusive trend in the results for CC plans for the smallest lesions, as the results varied regardless of size. The reason for this is likely related to isocenter placement. In HD120 MLC plans, the isocenter is placed at the PTV mass center for small PTVs, making it less susceptible to small movements. In contrast, cones cannot be placed at the mass center in cone planning, as they must be positioned manually to cover the PTV as well as possible.

In the context of HD120 MLC and small volumes, a potential issue is a deviation in the leaves’ positions that may occur, which can be significant compared to the size of the PTV. In this case, a cone may be a better option due to its mechanical stability. However, measurements have shown that both ECDC and AcurosXB can be trusted when dealing with small volumes in a homogenous medium.

QA plans without couch rotations using gamma analysis resulted in a 100% passing rate for CC plans. In contrast, HD120 MLC plans resulted in slightly lower passing rates of 98.76% and 99.52% for the two PTVs with the lowest volumes. The measurements were repeated five times, and the results were consistent. The slight differences in results between collimators were due to the final dose calculation resolutions. During measurements, it was observed that lowering the dose calculation resolution from 0.6 mm to 1.25 mm resulted in a lower gamma analysis passing rate by a few percent.

The results of QA measurements with table rotations showed a slightly different trend. The passing rate decreased with an increase in PTV volume. For these measurements, PTVs were delineated 3 cm away from the central detector of the SRS MapCHECK, closer to the edge of the SRS Stereotactic Phantom. With higher PTV volumes, there is less space between the PTV edge and the phantom edge, but there is also a greater volume of lower doses around the PTV. As a result of the proximity of the phantom edge, some of the doses were not measured.

## 5. Conclusions

The conical collimator has been thought to allow steeper dose fall-off outside the PTV, potentially leading to better sparing of OARs. However, the results of this study indicate that this may not necessarily be the case for the Varian Edge linear accelerator. After planning 116 pairs of treatment plans for single-fraction heterogeneous doses, it was found that cones could produce results almost as good as HD120 MLC, and the true benefit of steeper dose fall-off is questionable.

Regarding steeper dose fall-off, CC only shows slightly better results in dose distributions outside the PTV for PTV volumes below 0.5 cm^3^. In these cases, cones do tend to spare the brain a little more than HD120 MLC, but other OARs do not show the same benefit. However, the question arises as to whether the differences of 0.3 cm^3^ and 0.7 cm^3^ for V10 Gy and V5Gy are clinically significant for patient treatments.

The incidence of radiation necrosis and swelling, among other side effects, increases with the size of the PTV. Therefore, large metastases are typically treated with 3–5 fractions instead of a single fraction. Given CC offers no advantage over HD120 MLC for larger lesions, CC will not be used. In cases with smaller lesions, where all dose constraints are well below critical values, the differences in the absorbed doses in the brain have no clinical benefit. Choosing a complex and lengthy treatment over a simpler and shorter one for questionable patient benefit is unrealistic.

The findings of this investigation provide evidence that the HD120 MLC can be relied upon to administer treatment to the smallest PTVs. When properly calibrated, the delivered dosage is as precise as CC’s.

A cone collimator is purchased separately, and before purchase, one should know what it brings to clinical practice and what would be the actual benefit for patients. Compared to HD120 MLC, this study shows that the true benefit for patients with brain metastases could be minimal at best. The CC could be an upgrade for SRS if the hospital has MLC with 0.5 mm leaf width or higher, but maybe a better choice for an upgrade would be HD120 MLC, as CC can only be used intracranially.

## Figures and Tables

**Table 1:** PTV D_98%_, median and D_2%_ doses in Gy are represented as mean values with standard deviations for all groups and collimators.

HD120 MLC				
	Group1	Group 2	Group 3	Group 4
D_98%_ (Gy)	25.73 ± 0.33	25.47 ± 0.91	26.05 ± 0.36	25.89 ± 0.52
D_MEDIAN_ (Gy)	29.83 ± 1.51	31.05 ± 1.06	33.69 ± 1.33	32.17 ± 1.04
D_2%_ (Gy)	33.64 ± 2.55	35.65 ± 2.28	38.54 ± 2.20	35.84 ± 1.66
**CC**				
D_98%_ (Gy)	26.64 ± 1.08	25.73 ± 3.06	26.78 ± 1.16	23.73 ± 1.61
D_MEDIAN_ (Gy)	32.44 ± 3.86	35.39 ± 4.86	31.65 ± 4.78	42.31 ± 10.48
D_2%_ (Gy)	35.96 ± 6.18	43.39 ± 5.97	32.58 ± 5.12	60.36 ± 6.18

**Table 2:** Volumes of isodose lines for 25 Gy, 12.5 Gy, 10 Gy and 5 Gy doses surrounding PTV: mean values with standard deviation.

HD120 MLC				
	Group1	Group 2	Group 3	Group 4
V_25Gy_ (cm^3^)	0.31 ± 0.13	0.81 ± 0.12	1.85 ± 0.58	4.51 ± 0.95
V_12.5Gy_ (cm^3^)	1.27 ± 0.39	2.62 ± 0.36	4.97 ± 1.50	12.25 ± 2.43
V_10Gy_ (cm^3^)	1.73 ± 0.49	3.81 ± 0.52	6.47 ± 1.82	16.64 ± 3.27
V_5Gy_ (cm^3^)	4.88 ± 1.41	10.71 ± 2.28	17.60 ± 4.95	44.85 ± 8.85
**CC**				
V_25Gy_ (cm^3^)	0.35 ± 0.16	0.93 ± 0.40	2.53 ± 0.78	4.69 ± 1.04
V_12.5Gy_ (cm^3^)	0.97 ± 0.40	2.80 ± 0.57	6.15 ± 1.69	12.66 ± 2.81
V_10Gy_ (cm^3^)	1.30 ± 0.55	3.81 ± 0.88	8.27 ± 2.37	17.62 ± 3.94
V_5Gy_ (cm^3^)	3.64 ± 1.53	11.34 ± 3.49	22.12 ± 6.71	54.91 ± 14.14

**Table 3:** PTV dose parameters for both collimators across groups. Mean values with standard deviation.

HD120 MLC				
	Group1	Group 2	Group 3	Group 4
CI	1.12 ± 0.24	1.11 ± 0.16	1.09 ± 0.59	1.07 ± 0.02
HI	1.35 ± 0.99	1.43 ± 0.09	1.54 ± 0.09	1.36 ± 0.67
DGI	4.44 ± 1.02	3.12 ± 0.25	2.70 ± 0.21	2.73 ± 0.15
PI	0.92 ± 0.14	0.93 ± 0.02	0.92 ± 0.05	0.95 ± 0.02
**CC**				
CI	1.19 ± 0.28	1.30 ± 0.34	1.52 ± 0.33	1.11 ± 0.89
HI	1.44 ± 0.25	1.74 ± 0.56	1.30 ± 0.21	1.91 ± 0.25
DGI	2.86 ± 0.85	3.01 ± 0.21	2.46 ± 0.18	2.70 ± 0.09
PI	0.89 ± 0.25	0.83 ± 0.07	0.81 ± 0.14	0.90 ± 0.06

**Table 4:** Mean values with standard deviations of absorbed doses in the brain for both collimators in all groups.

HD120 MLC				
	Group1	Group 2	Group 3	Group 4
V_5Gy_ (cm^3^)	4.54 ± 1.39	9.23 ± 2.04	12.19 ± 5.25	34.83 ± 7.97
V_10Gy_ (cm^3^)	1.63 ± 0.49	3.39 ± 0.69	4.03 ± 1.76	11.79 ± 2.22
**CC**				
V_5Gy_ (cm^3^)	3.2 ± 1.34	10.15 ± 3.96	15.47 ± 6.99	41.42 ± 11.34
V_10Gy_ (cm^3^)	1.09 ± 0.46	3.43 ± 0.88	5.36 ± 2.38	12.20 ± 2.39

**Table 5:** Median values of maximum doses for every OAR for both collimators across all groups.

HD120 MLC				
	Group1	Group 2	Group 3	Group 4
Brainstem	0.16	0.35	1.15	1.93
Eye Right	0.08	0.05	0.21	0.26
Eye Left	0.07	0.14	0.18	0.35
Lens Left	0.02	0.02	0.07	0.16
Lens Right	0.02	0.01	0.07	0.15
Opticus Left	0.06	0.07	0.25	0.41
Opticus Right	0.08	0.06	0.18	0.36
Chiasm	0.10	0.19	0.55	0.46
**CC**				
Brainstem	0.20	0.56	1.02	2.33
Eye Right	0.15	0.17	0.29	0.50
Eye Left	0.14	0.12	0.28	0.48
Lens Left	0.06	0.02	0.14	0.01
Lens Right	0.05	0.04	0.15	0.01
Opticus Left	0.10	0.17	0.27	0.30
Opticus Right	0.13	0.14	0.31	0.34
Chiasm	0.20	0.33	0.48	1.78

**Table 6:** Lowest PTV coverage differences and treatment plan pairs comparisons for both collimators across group 1.

**Lowest PTV Coverage Differences**				
		**Minimum**	**Maximum**	**Mean**
PTV coverage (%)		79.90	99.79	94.44 ± 5.55
			CC	
PTV coverage (%)		73.26	95.79	87.70 ± 4.95
**Treatment plan pairs comparisons (group 1, 35 pairs)**				
			**HD120 MLC**	**CC**
Number of plans with higher lowest PTV coverage			28	7
Highest PTV coverage difference between pairs			25.97%	4.41%
Number of plans with lowest PTV coverage over 95%			20	1
Number of plans with lowest PTV coverage between 90% and 94.99%			0	8
Number of plans with lowest PTV coverage below 90%			15	26

**Table 7:** Measurements without couch rotations: gamma analysis passing rate for both collimator.

PTV Diameter (mm)	HD120 MLC	CC
4	98.76%	100%
6	99.52%	100%
**8**	100%	100%
10	100%	100%
12	100%	100%
15	100%	100%
17	100%	100%

**Table 8:** Measurements with couch rotations. Gamma analysis passing rate for both collimators.

PTV Volume (cm^3^)	HD120 MLC	CC
0.03	100%	100%
0.18	98.4%	100%
0.73	95.5%	99.1%

## Data Availability

The data presented in this study is stored on repository at Radiochirurgia Zagreb and are available on request from the corresponding author.
